# An investigation into the correlation between intraperitoneal teicoplanin concentrations and treatment outcomes in peritoneal dialysis-associated peritonitis

**DOI:** 10.3389/fphar.2024.1446774

**Published:** 2024-09-09

**Authors:** Lulu Wang, Jiangqing Fan, Xuejie Chen, Wenpu Lei, Chunming Jiang, Hang Liu, Yun Yang, Jizhong Shen

**Affiliations:** ^1^ Department of Pharmacy, Nanjing Drum Tower Hospital, Affiliated Hospital of Medical School, Nanjing University, Nanjing, China; ^2^ Hunan Provincial Key Laboratory of the Research and Development of Novel Pharmaceutical Preparations, The “Double-First Class” Application Characteristic Discipline of Hunan Province (Pharmaceutical Science), Changsha Medical University, Changsha, China; ^3^ Department of Pharmacy, Nanjing Drum Tower Hospital, School of Basic Medicine and Clinical Pharmacy, China Pharmaceutical University, Nanjing, China; ^4^ Department of Nephrology, Nanjing Drum Tower Hospital Clinical College of Traditional Chinese and Western Medicine, Nanjing University of Chinese Medicine, Nanjing, China; ^5^ Department of Stomatology, Nanjing Drum Tower Hospital, Affiliated Hospital of Medical School, Nangjing University, Nangjing, China

**Keywords:** peritoneal dialysis-associated peritonitis, teicoplanin, peritoneal dialysis effluent, therapeutic drug monitoring, individualized drug therapy

## Abstract

Peritoneal dialysis-associated peritonitis (PDAP) is a frequent complication of peritoneal dialysis. The guidelines from the International Society for Peritoneal Dialysis (ISPD) suggest administering teicoplanin through the peritoneal route to treat PDAP, but do not specify the ideal concentration for peritoneal dialysis effluent (PDE). Patients meeting the trial criteria for PDAP in our hospital between July 2022 and December 2023 were enrolled. Data on PDE white blood cell count, PDE neutrophil percentage, clinical symptoms, CRP, and PCT were gathered pre- and post-treatment. Incidences of adverse drug reaction (ADR) and case numbers during treatment were recorded. Subsequently, patients were categorized into cured and uncured groups for evaluating the relationship between PDE teicoplanin concentration and treatment effectiveness. The self-control study results on teicoplanin efficacy indicated intraperitoneal teicoplanin administration achieved an efficacy rate of 88.9% and an ADR incidence of 5.5% in treating PDAP patients. There was no observed correlation between teicoplanin blood concentration and PDE concentration. PDE teicoplanin concentrations on days 1, 3, 5, and 7 post-dosing were higher inthe cured group, with a significant contrast in PDE concentration on day 5 between the 18.98 ± 2.43 mg/L of the cured group and the 12.07 ± 2.68 mg/L of the uncured group. ROC curve revealed a higher likelihood of cure in patients when PDE teicoplanin concentration exceeded 15.138 mg/L on day 5 post-dosing. Univariate and multifactorial studies identified 24-h urine volume and the number of daily abdominal dialysis sessions as influential factors in PDE teicoplanin concentration on day 5. A positive correlation was found between 24-h urine volume and PDE teicoplanin concentration, with PDAP patients having urine volume over 537 mL showing significantly higher drug concentrations. Conversely, the number of daily PDAP sessions was negatively correlated with PDE teicoplanin concentrations, indicating that patients with 1∼3 daily PDAP sessions had notably higher PDE teicoplanin concentrations compared to those with 4∼6 sessions. Therefore, PDAP patients who use intraperitoneal teicoplanin could effectively control infection by monitoring the PDE teicoplanin concentration (>15.138 mg/L) on day 5 after dosing.

## Introduction

Peritoneal dialysis (PD) is a clinical method used for treating patients with end-stage renal disease (Sahinoz et al., 2020). PD-associated peritonitis (PDAP) is a common complication in patients undergoing continuous ambulatory PD. This complication is mainly caused by the long-term placement of short dialysis tubing in the peritoneal cavity and the daily fluid changes, which can lead to PDAP. Patients with end-stage renal disease often have other comorbidities that significantly raise the risk of peritonitis (Catar et al., 2017; Cetin et al., 2017). PDAP is the main reason for switching PD patients to hemodialysis. Prolonged peritonitis can result in peritoneal failure and, ultimately, death (Cho and Struijk, 2017). The occurrence of PDAP can disrupt the patient’s daily PD routine, leading to increased hospitalization and mortality rates (Lee et al., 2019).

The International Society for PD (ISPD) recommends using glycopeptides (vancomycin or teicoplanin) as the initial empiric treatment for patients infected with gram-positive bacteria (Li et al., 2022). Vancomycin has drawbacks such as increased risk of G^+^ resistance and adverse reactions like ototoxicity and nephrotoxicity, but teicoplanin offers a clear advantage (Neville et al., 1988a). Therefore, teicoplanin is widely used in clinical practice for empiric treatment of G^+^ bacterial infections in PDAP patients. The therapeutic efficacy of teicoplanin is primarily assessed based on the trough concentration value (C_min_) through therapeutic drug monitoring. However, the ISPD guidelines do not specify the target peritoneal dialysis effluent (PDE) teicoplanin concentration for intraperitoneal administration. The guidelines mainly rely on foreign literature with limited direct clinical research evidence for application to PDAP patients in China. Some reports indicate that teicoplanin blood concentration in PDAP patients may not accurately predict treatment outcomes (Neville et al., 1988a; Deacon et al., 2023; Blunden et al., 2007). This study aims to investigate the concentration distribution of PDE teicoplanin in the Chinese population, analyze the correlation between teicoplanin concentrations in peritoneal effluent and serum, explore factors influencing PDE drug concentration, and enhance the application data for intraperitoneal teicoplanin administration in PDAP patients in China.

## Materials and methods

### Research subjects

This study was a single-center research project conducted at the Nephrology Department of Nanjing Drum Tower Hospital. The study followed the “Helsinki Declaration” and obtained approval from the Medical Ethics Committee of Nanjing Drum Tower Hospital (Approval No: 2022-003-02). Patients were informed about the research objectives, precautions, risks, and other relevant aspects of the study before enrollment, through oral and written communication.

**FIGURE 1:**
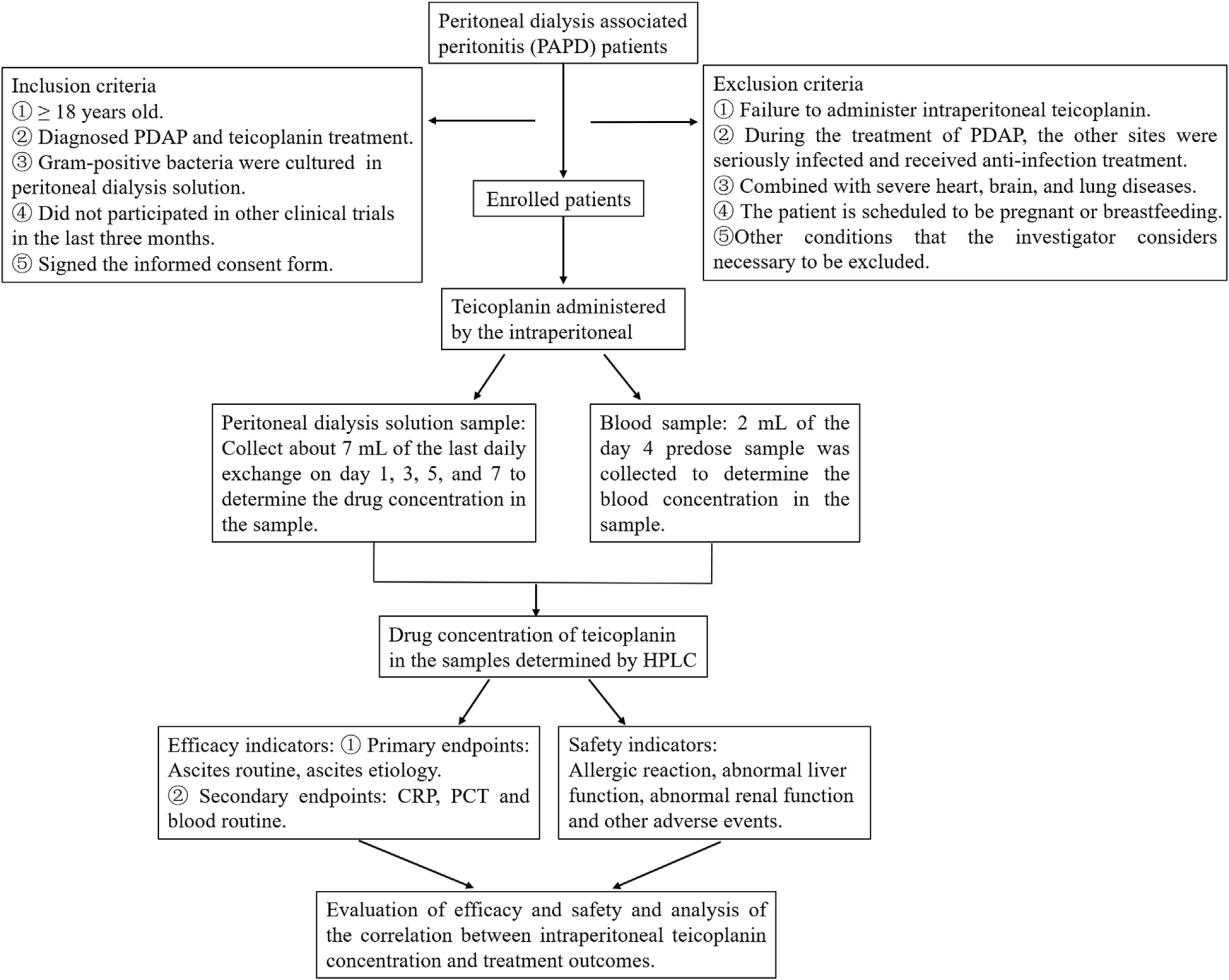
The flowchart to evaluate the intraperitoneal administration of teicoplanin for PDAP.

According to the ISPD guidelines recommendation (Li et al., 2022), doctors can establish a diagnosis of PDAP. The study’s inclusion criteria require participants to be 18 years of age or older, have a clinical diagnosis of PDAP, and receive treatment through intraperitoneal administration of teicoplanin, with gram-positive bacteria cultured from PD effluent. Participants should not have taken part in other clinical trials in the past 3 months, understand the trial’s purpose, willingly participate, and sign informed consent forms. Exclusion criteria include patients who did not receive intraperitoneal teicoplanin throughout their treatment, those who developed severe infections at other sites during PDAP treatment and received antimicrobial therapy, individuals with severe heart, brain, and lung diseases combined, patients planning pregnancy or currently breastfeeding, and any other conditions deemed necessary to exclude by the researchers (Figure 1).

### Treatment plan

The injectable teicoplanin, with a specification of 0.2 g per vial (200,000 units), is the medication used for treatment. It is manufactured by Zhejiang Medicine Co., Ltd. The treatment protocol of this study chose a continuous infusion of teicoplanin with a daily dose of 0.2 g per patient based on the ISPD guidelines (Li et al., 2022). According to the patient’s PD plan, teicoplanin for injection was evenly distributed into each bag of PD fluid before the patient’s PD procedure to ensure that each bag of dialysis fluid entering the peritoneal cavity contained teicoplanin. When the number of daily dialysis sessions is even, If a patient required four PD procedures daily, the 0.2 g dose was divided into four equal parts, with 0.05 g of dissolved teicoplanin added to the PD fluid before each procedure, followed by the exchange of dialysis fluid. When the number of dialysis sessions per day is odd, For example, on peritoneal dialysis three times a day, we dissolved 0.2 g teicoplanin in 3 mL normal saline, then added 1 mL teicoplanin to 2 L peritoneal dialysis fluid, poured 2 L peritoneal dialysis fluid containing 1 mL teicoplanin into the patient’s abdominal cavity for 4 h, and repeated this operation three times. The number of dialysis times per day is determined by the peritoneal function of each peritoneal dialysis patient.

### The end point of research and related definitions

After 7 days of treatment, all patients underwent a assessment to evaluate the effectiveness. This assessment considered primary efficacy endpoints, secondary efficacy endpoints, and primary safety endpoints. The primary efficacy endpoints in this study are: 1) White blood cell count in PD effluent (p-WBC) < 100 × 10^6^/L; 2) Percentage of neutrophilic granulocytes in PD effluent (p-NE%) < 50%; 3) Resolution of abdominal pain, absence of fever, and clarity of PD effluent in clinical signs of patients. According to ISPD guidelines, meeting all three criteria or two of the three constitutes a cure, while meeting one is classified as partial relief. The efficacy calculation involves determining the proportion of cured and partially relieved patients among the total.

The secondary efficacy endpoints of this study mainly involve infectious markers: C-reactive protein (CRP) and procalcitonin (PCT); Hematological parameters: white blood cell count (b-WBC), blood neutrophilic granulocyte percentage (b-NE%), and hemoglobin (Hb); Renal function markers: serum creatinine (Scr), urea, uric acid (UA), and albumin (ALB).

The study’s primary safety outcome is the incidence of adverse events, such as allergic reactions, hematologic adverse reactions like leukopenia, and hepatic dysfunction. Adverse event incidence is calculated as the percentage of adverse events in the study group relative to the total number of patients in that group. Nephrotoxicity is defined as a 150% increase in serum creatinine levels from baseline, while hepatotoxicity is defined as aspartate aminotransferase (AST) or alanine aminotransferase (ALT) levels exceeding three times the upper limit of normal (Ueda et al., 2016).

### Sample collection and preprocessing

All patients received peritoneal dialysate and serum samples. Peritoneal dialysate samples were collected on the first, 3rd, 5th, and 7th days of teicoplanin administration. Serum samples were collected on the 4th day prior to drug administration. Blood samples were centrifuged at 3,000 rpm for 10 minutes. The upper serum layer was then transferred into labeled EP tubes and stored at −80°C. Peritoneal fluid samples were collected daily after the PD procedure. The peritoneal fluid bag was disinfected with iodine, and peritoneal effluent was withdrawn using a sterile syringe and stored at −80°C.

### Sample concentration determination method

High-performance liquid chromatography (HPLC) was employed to analyze the teicoplanin concentration in the PD effluent. An Agilent Zorbax SB-C18 column (4.6 × 250 mm, 5 μm) was used. Methyl p-hydroxybenzoate served as the internal standard. The column temperature was kept at 35°C. The mobile phase comprised acetonitrile - 0.01 mol/L potassium dihydrogen phosphate (26:74, v/v, pH = 2.8 adjusted by phosphoric acid). A flow rate of 1 mL/min was set, with an injection volume of 40 μL. UV detection was conducted at 215 nm, while the column temperature remained at 40°C. The pretreatment and chromatographic methods for detecting teicoplanin in serum and PD effluent are the same (Supplement Material).

### Statistical methods

SPSS 26.0 software analyzed all data. The independent samples t-test was used for normally distributed variables, while the rank sum test was used for non-normally distributed variables. Count data were presented as cases, and the chi-square test was used. Pearson correlation analysis was applied to normally distributed variables, while spearman analysis was used for non-normally distributed variables. A significance level of *P* < 0.05 indicated statistical significance. A pearson correlation coefficient (*r*) greater than 0 indicated a positive correlation. GraphPad Prism 8.0.2 was used for data plotting.

## Results

### Basic information

From July 2022 to December 2023, a total of 36 patients with PDAP were enrolled in the Department of Nephrology at Nanjing Drum Tower Hospital. Based on the treatment outcomes of patients, 36 cases of PDAP patients who received intraperitoneal teicoplanin were divided into a “Total group”, a “Cured” group and an “Uncured” group (Table 1). Basic patient information comparison, including age, gender, BMI, and PD age, showed no statistically significant differences between the groups. Laboratory test results before treatment, such as p-WBC, p-NE%, b-WBC, CRP, and PCT, also displayed no significant variations between the two groups except 24-h urine volume and PD frequency.

**TABLE 1:** Basic information of 36 PDAP patients.

Project	Total group (n = 36)	Cured group (n = 29)	Uncured group (n = 7)	*P*-value
Age (years)	60.30 ± 15.18	60.62 ± 14.17	59.00 ± 20.12	0.231
Male/case (%)	22 (61)	17 (58.6)	5 (71.4)	0.289
Systolic pressure (mmHg)	129.08 ± 25.81	132.38 ± 19.29	137.89 ± 21.32	0.562
Diastolic pressure (mmHg)	82.83 ± 19.49	97.38 ± 13.29	88.39 ± 10.12	0.382
Body mass index (kg/m^2^)	23.67 (20.81, 28.71)	25.21 ± 8.03	27.75 ± 7.29	0.128
PD age (months)	23.5 (9.25, 51.75)	24.00 (11.00, 52.00)	21.00 (3.00, 46.00)	0.671
24-h urine volume (mL)	200 (0, 537.5)	573.28 (452.78, 1393.98)	239.39 (159.23, 489.21)	0.028*
p-WBC (*106/L)	1333.5 (573.50, 4475.00)	1330.00 (549.00, 4833.50)	1830.00 (691.00, 4388.00)	0.452
p-NE%	84.50 (70.90, 90.15)	84.80 (68.05, 89.85)	83.60 (75.10, 94.40)	0.124
b-WBC (*109/L)	6.40 (5.32, 8.00)	7.16 ± 3.35	8.94 ± 4.19	0.098
b-NE%	77.60 (67.42, 84.95)	74.65 ± 9.90	78.01 ± 23.38	0.355
CRP (mg/L)	37.50 (10.00, 101.27)	26.50 (6.20, 74.85)	35.90 (7.10, 112.60)	0.189
PCT (ng/mL)	0.66 (0.30, 2.99)	0.55 (0.28, 2.78)	0.72 (0.38, 16.55)	0.236
Scr (μmol/L)	772.45 ± 320.15	755.69 ± 307.84	841.90 ± 385.60	0.293
PD frequency (times)	4.36 ± 2.29	2.6 ± 1.42	5.28 ± 1.04	0.043*

Note: Data are described as means ± standard deviation or mean (Q25, Q75). * indicates statistical difference *P* < 0.05, ** indicates statistical difference *P* < 0.01.

### The efficacy of the teicoplanin before and after treatment in PDAP patients

When gram-positive bacterial infections are treated with intraperitoneal teicoplanin, the standard treatment duration is generally 2 weeks. However, most patients show significant improvement in clinical symptoms within 1 week. In this study, primary endpoints were evaluated based on routine PD effluent analysis and clinical symptom assessment after 7 days of teicoplanin administration, and secondary endpoint were evaluated to CRP, PCT and blood routine after 14 days of teicoplanin administration. Out of the patients, 29 were cured, 3 were in partial remission, and 4 experienced treatment failure (1 patient died, and 3 patients permanently transitioned to hemodialysis), resulting in an overall treatment success rate of 88.9%.

PDAP patients not treated with teicoplanin were classified as the pre-treatment group, while those who received teicoplanin were in the post-treatment group. For primary endpoints, the white blood cell count in peritoneal effluent was as high as 1333.5 (573.50, 4475.00) × 10^6^/L before treatment, decreasing to 28.00 (10.25, 75.00) × 10^6^/L after treatment, showing a statistically significant difference (Table 2). Additionally, after treatment, the neutrophil percentage in PD effluent decreased significantly [84.50 (70.90, 90.15) % vs 25.00 (13.55, 48.95) %], and the lymphocyte count in PD effluent dropped from 196.00 (91.15, 444.62) × 10^6^/L to 14.00 (6.25, 49.75) × 10^6^/L (*P* < 0.05). Following teicoplanin treatment, the number of patients experiencing abdominal pain, diarrhea, fever, and cloudy PD fluid notably decreased, particularly evident in the disappearance of abdominal pain and cloudy PD fluid symptoms in many patients.

**TABLE 2:** Comparison of effectiveness observations.

Key observations indicators	Pre-treatment group (n = 36)	Post-treatment group (n = 36)	*P*-value
p-WBC (*10^6^/L)	1333.5 (573.50, 4475.00)	28.00 (10.25, 75.00)	<0.01**
p-NE (*10^6^/L)	1099.60 (418.57, 3333.62)	4.00 (1.00, 18.00)	<0.01**
p-NE%	84.50 (70.90, 90.15)	25.00 (13.55, 48.95)	<0.01**
p-Lym (*10^6^/L)	196.00 (91.15, 444.62)	14.00 (6.25, 49.75)	<0.01**
p-Lym%	14.55 (9.85, 29.1)	72.85 (45.95, 86.45)	<0.01**
Abdominal pain	31 (86.1%)	3 (8.3%)	<0.01**
Diarrhea	9 (25%)	1 (2.7%)	0.014*
Fever	8 (22.3%)	1 (2.7%)	0.028*
Peritoneal dialysate turbidity	26 (72.2%)	2 (5.6%)	<0.01**
Secondary indicators			
CRP (mg/L)	37.50 (10.00, 101.27)	8.30 (3.55, 19.33)	0.002**
PCT (ng/mL)	0.66 (0.30, 2.99)	0.61 (0.29, 1.15)	0.480
b-WBC (*10^9^/L)	6.40 (5.32, 8.00)	6.20 (5.32, 8.85)	0.690
b-NE%	77.60 (67.42, 84.95)	69.10 (59.32, 72.60)	0.005**

Note: Data are described as mean (Q25, Q75). * indicates statistical difference *P* < 0.05, ** indicates statistical difference *P* < 0.01.

For secondary endpoints, the CRP level decreased significantly from 37.50 (10.00, 101.27) mg/L to 8.30 (3.55, 19.33) mg/L post-treatment (Table 2). Conversely, there was only a slight change in PCT, which was not statistically significant. Following treatment, a notable decrease in the neutrophil-to-lymphocyte ratio was observed, showing a statistically significant difference compared to pre-treatment levels. However, the variance in serum white blood cell count before and after treatment was minimal and not statistically significant.

### Drug safety

During the treatment process, two cases of ADR were observed. The first patient developed an allergic reaction with skin itching on the third day of teicoplanin treatment. The second patient’s alanine transaminase level rose to 133 U/L (above 120 U/L) while undergoing teicoplanin therapy. In both instances, the ADR-related symptoms normalized upon discontinuation of teicoplanin or provision of symptomatic treatment. Another patient exhibited fever after 3 days of teicoplanin administration, later attributed to PDAP, ruling out teicoplanin-induced drug fever. Consequently, the incidence of teicoplanin ADR in this study was 5.5%.

### PDE teicoplanin concentration and PDAP patients outcomes

This research established an effective method to measure the teicoplanin concentration in peritoneal dialysis effluent (PDE) using HPLC (Supplementary Figures S1, S2). The Pearson correlation analysis showed no significant correlation between serum teicoplanin concentration and PDE teicoplanin concentration on the first, 3rd, 5th, and 7th days post-medication. Essentially, serum teicoplanin concentration cannot accurately reflect or be converted to the corresponding teicoplanin concentration in the abdominal cavity (Supplementary Table S1).

A comparison of major serum indicators and PDE markers in the cured group and the uncured group revealed significant differences after intraperitoneal administration of teicoplanin. The results indicated that the p-WBC of the cured group was 15.00 (8.00, 45.00) × 10^6^/L, while in the uncured group, the p-WBC was 159.00 (112.5, 1023.5) × 10^6^/L. The p-WBC of the cured group decreased to the normal range and was significantly lower than that in the uncured group. There was also a significant difference in p-NE% between the two groups. Other indicators such as b-WBC, b-NE%, CRP, PCT, and serum teicoplanin concentration did not show significant differences between the two groups (Table 3).

**TABLE 3:** The association between PDE teicoplanin concentration and PDAP patient outcomes.

Laboratory examination after treatment	Cured group	Uncured group	*P*-value
p-WBC (*10^6^/L)	15.00 (8.00, 45.00)	159.00 (112.5, 1023.5)	0.001**
p-NE%	27.01 ± 19.08	58.86 ± 33.91	0.005**
b-WBC (*10^9^/L)	6.92 ± 2.47	6.40 ± 2.28	0.641
b-NE%	65.39 ± 9.85	71.08 ± 25.08	0.607
Serum teicoplanin concentration	11.64 ± 7.74	10.85 ± 4.92	0.801
CRP (mg/L)	6.27 (3.13, 12.10)	34.00 (5.20, 69.38)	0.053
PCT (ng/mL)	0.57 (0.27, 1.12)	0.66 (0.29, 2.87)	0.665
Day 1 PDE teicoplanin concentration (mg/L)	13.65 ± 3.26	11.48 ± 3.39	0.469
Day 3 PDE teicoplanin concentration (mg/L)	14.17 (6.80, 22.93)	20.38 (11.72, 23.75)	0.299
Day 5 PDE teicoplanin concentration (mg/L)	18.98 ± 2.43	12.07 ± 2.68	<0.01**
Day 7 PDE teicoplanin concentration (mg/L)	20.89 (9.45, 30.23)	11.43 (8.42, 13.21)	0.061

Cured group (n = 29), Uncured group (n = 7). Data are described as means ± standard deviation or mean (Q25, Q75). * indicates statistical difference *P* < 0.05, ** indicates statistical difference *P* < 0.01.

After intraperitoneal administration of teicoplanin, there were no significant differences in serum teicoplanin concentrations between the two groups on days 1, 3, and 7. However, the PDE teicoplanin concentration in the cured group was 18.98 ± 2.43 mg/L on the 5th day after treatment, while it was 12.07 ± 2.68 mg/L in the uncured group. This study showed a notable variation in PDE teicoplanin concentrations and compliance rates (Supplementary Table S2) between the “Cured” group and the “Uncured” group by the 5th day of treatment.

### ROC analysis of PDE teicoplanin concentration and treatment outcome on day 5

**FIGURE 2:**
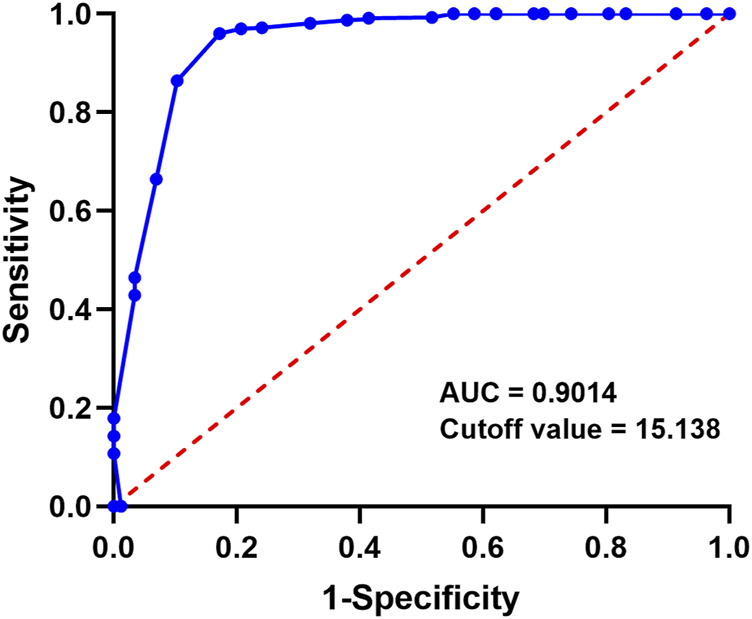
Day-5 PD teicoplanin concentration versus ROC curve of patient survive.

To investigate if the teicoplanin concentration in the abdominal cavity on the 5th day after medication affects patient outcomes, the ROC curve assessed the predictive effect of the 5th day PDE concentration on patient recovery. The results showed an area under the ROC curve of 0.9014 (95% CI: 0.8313–1.000), indicating a strong predictive effect of the 5th day PDE teicoplanin concentration on patient recovery (Figure 2). The Youden index was 0.9026, with a cutoff value of 15.138. This suggests that patients are more likely to achieve a curative treatment outcome when the 5th day PDE teicoplanin concentration exceeds 15.138 mg/L after intraperitoneal teicoplanin administration.

### Analysis of factors affecting the PDE teicoplanin concentration in PDAP patients

**FIGURE 3:**
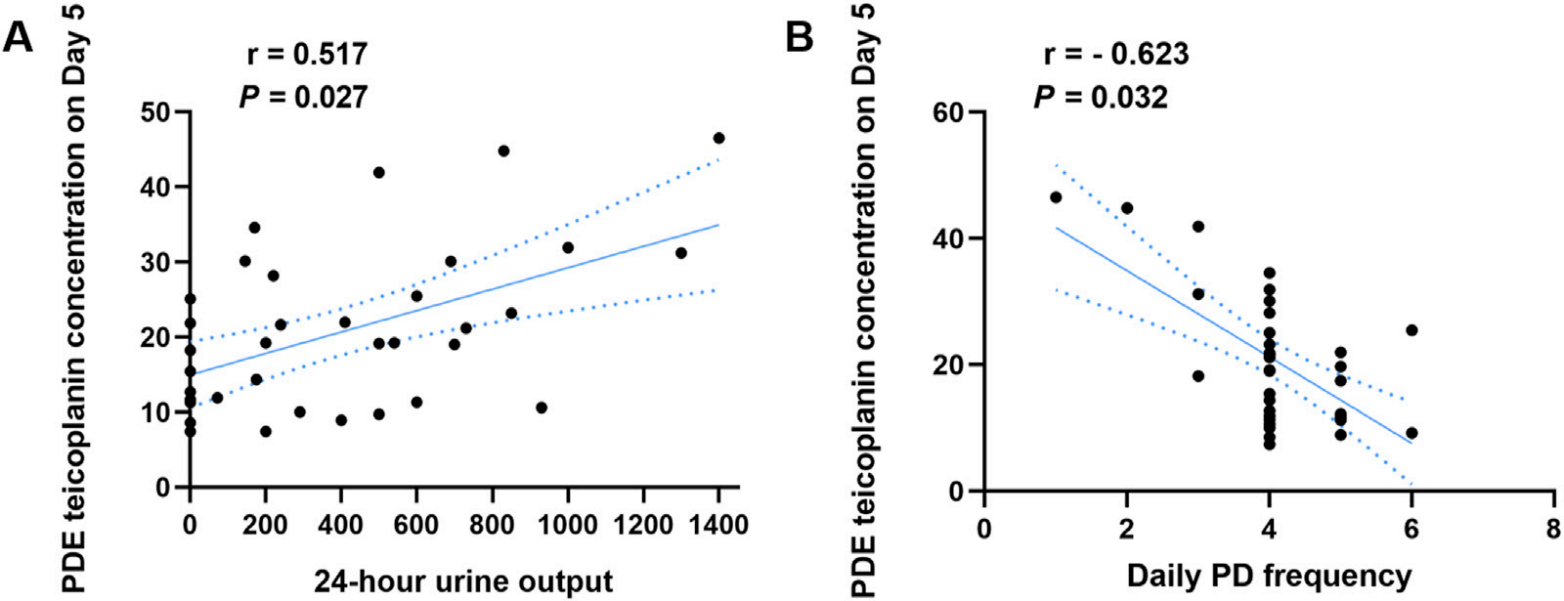
Correlation analysis between the PDE teicoplanin concentration and 24-h urine output or different daily PD frequency. **(A)** PDE teicoplanin concentrations and 24-h urine output. **(B)** PDE teicoplanin concentrations and daily PD frequency.

Single-factor analysis revealed that age, gender, body weights, properties of dialysate, residual renal function, and fibrinogen content did not affect the PDE teicoplanin concentration (Supplementary Table S3–S10). The 24-h urine output and daily PD frequency correlated with the PDE teicoplanin concentration. PDAP patients with a 24-h urine volume over 537 mL showed significantly higher teicoplanin concentrations than patients with volumes between 0 and 200 mL and 201–537 mL (Table 4). A Pearson correlation analysis indicated a correlation coefficient (*r*) of 0.517 with a significance level of *P* < 0.05 (Figure 3A).

**TABLE 4:** The relationship between the PDE teicoplanin concentration on Day 5 and 24-h urine output.

Grouping by 24-h urine volume	PDE teicoplanin concentration (mg/L) M50 (M25, M75)
1.0 (*n* = 17)	12.85 (8.12, 21.23)
2.0 (*n* = 10)	10.17 (7.38, 24.59)
3.0 (*n* = 9)	23.16 (18.89, 39.45)
*F*	6.378
*P*-value	0.039*

Note: *P < 0.05, **P < 0.01, 1 represents 0–200 mL, 2 represents 201–537 mL, and 3 represents >537 mL.

PDE teicoplanin concentrations in PDAP patients with 13 peritoneal dialysis per day were notably elevated compared to the other two groups (Table 5). Pearson correlation analysis indicated a correlation coefficient of −0.623 and *P* < 0.05, demonstrating a substantial negative association (Figure 3B).

**TABLE 5:** The relationship between the PDE teicoplanin concentration on Day 5 and daily PD frequency.

Grouped by daily PD dialysis frequency	Teicoplanin concentration (mg/L) (‾χ ± SD)
1.0 (*n* = 5)	38.71 ± 16.36
2.0 (*n* = 18)	12.89 ± 7.28
3.0 (*n* = 13)	17.67 ± 10.23
*F*	10.154
*P*-value	0.001**

Note: *P < 0.05, **P < 0.01, 1 represents 1–3 times, 2 represents 4 times, and 3 represents 5–6 times.

Multifactor analysis revealed that both 24-h urine volume (*F* = 3.974, *P* = 0.029) and daily PD frequency (*F* = 13.747, *P* < 0.01) exhibited significant differences, indicating the presence of main effects. This suggests that both the 24-h urine volume and daily PD frequency in patients greatly impact teicoplanin concentration in PDE on the 5th day post-medication (Table 6).

**TABLE 6:** Multivariate analysis of PDE teicoplanin concentration on day 5 post-administration.

Differential source	Sum of squares	Degree of freedom	Mean square	*F*	*P*-value
Intercept term	13621.142	1	13621.142	165.478	0.000**
24-h urine output	532.427	2	266.213	3.974	0.029*
Daily PD dialysis frequency	1841.649	2	920.824	13.747	0.000**
Standard error	2569.562	31	82.889		
Total	18564.780	36			

Note: *R* ^*2*^: 0.557, *P < 0.05, **P < 0.01.

## Discussion

For a long time, most evidence-based research on teicoplanin for PDAP has originated mainly from North America, Europe, and other regions (Neville et al., 1988b; Bonati et al., 1987). Although the treatment experience from these areas may not directly apply to patients in China, we need to actively explore customized treatment strategies for Chinese patients. This study focuses on examining the effectiveness and safety of intraperitoneal teicoplanin for PDAP, studying the relationship between teicoplanin concentrations in peritoneal dialysis fluid and treatment results, and identifying factors affecting individual responses. Our results show that intraperitoneal teicoplanin administration achieved an efficacy rate of 88.9% and an adverse drug reaction incidence of 5.5% in treating PDAP patients, using a self-before-and-after comparative approach at our peritoneal dialysis center.

Although a few related studies have been conducted in China in recent years (Xu et al., 2017), none have directly established the relationship between intraperitoneal teicoplanin concentrations and treatment outcomes in PDAP. The target teicoplanin concentration of 10 mg/L in the blood aims to prevent drug toxicity but does not indicate if the drug has reached therapeutic levels in the PDAP patient’s peritoneal cavity (Fish et al., 2012). Moreover, the correlation analysis revealed that serum teicoplanin concentration is not a reliable indicator of PDE teicoplanin concentrations. This study revealed a significant difference in PDE teicoplanin concentrations and compliance rates (Supplementary Table S2) between the “Cured” group and the “Uncured” group as early as the 5th day of treatment. The PDE teicoplanin concentration in the cured group on Day 5 was 18.98 ± 2.43 mg/L, significantly higher than the minimum inhibitory concentration for Gram-positive bacteria (4 mg/L) (Fish et al., 2012). ROC curve analysis indicated that if the PDE drug concentration on the 5th day exceeds 15.138 mg/L, PDAP patients are more likely to be cured. The results suggest that the PDE teicoplanin concentration on the 5th day (15.138 mg/L) strongly predicts the treatment outcome of PDAP patients.

Some studies indicated that the glomerular filtration rate and age can impact the blood concentration of teicoplanin 72 h after intravenous administration (Pea et al., 2003; Hu et al., 2018). However, Roberts and Byrne argued that serum albumin independently affected the serum concentration of teicoplanin (Roberts et al., 2014; Byrne et al., 2017). This research is the first to explore the factors influencing the PDE concentration of teicoplanin on the 5th day after intraperitoneal administration in PDAP patients. The one-way ANOVA analysis demonstrated that the 24-h urine output and daily PD frequency could influence the concentration of teicoplanin in the PDE. In simple terms, higher 24-h urine output correlates with higher PDE teicoplanin concentration in patients. Hiramatsu’s research found no significant difference in 24-h urine output among PD patients of different ages (Hiramatsu et al., 2012), suggesting that the study’s conclusion is applicable to PDAP patients across all age groups. Jin Sanli et al. discovered that an increased frequency of daily PD sessions is associated with reduced overall quality of life and physiological function in patients (Jin and Lu, 2010). The findings of this study indicated that a higher daily PD frequency was linked to lower PDE concentration. For the patients in the uncured group who experienced resistance to PDAP, our analysis of teicoplanin concentrations in peritoneal dialysate on days 5 and 7 indicates that low teicoplanin levels may have influenced treatment outcomes. Furthermore, multifactorial ANOVA reveals that 24-h urine output and dialysis frequency are the main factors affecting teicoplanin concentration in the dialysate. By examining the clinical information of seven patients in the uncured group on days 5 and 7, we found that in four cases, urine output significantly decreased on day 3 of teicoplanin therapy, while the other three exhibited edema symptoms and increased dialysis frequency. Thus, the reasons for the lack of cure in these seven patients confirmed that 24-h urine output and dialysis frequency affected the therapeutic outcomes of intraperitoneal teicoplanin administration.

This research has some limitations, like a small sample size and short study duration. To produce more conclusive results and support these findings, further investigation is necessary through clinical research with larger sample sizes and longer follow-up. Additionally, this study is the first to look at factors influencing the use of teicoplanin catheters in the peritoneal cavity among PDAP patients, with only 24-h urine volume and daily PD frequency as identified factors. Future research should involve large-scale clinical studies across multiple centers to examine additional influencing factors and patterns. Also, more time points are needed for collecting PD fluid samples in future studies, including intervals like 30 min, 1 h, 2 h, and 6 h after drug administration for a more thorough evaluation of teicoplanin’s pharmacokinetic characteristics with intraperitoneal administration. This method will aid in studying population pharmacokinetic (PPK) parameters and models for intraperitoneal teicoplanin administration in PDAP patients, leading to the development of more precise personalized dosing plans.

In conclusion, this study has elucidated the effectiveness and safety of intraperitoneal teicoplanin administration in PDAP patients. It has established a reliable and efficient HPLC methodology for quantifying teicoplanin concentrations. Furthermore, it has determined the optimal critical value for PDE teicoplanin concentration, which is 15.138 mg/L. The study has identified that the 24-h urine volume and the frequency of PD exchanges per day are the primary influencing factors on the PDE teicoplanin concentrations of the fifth day following intraperitoneal administration. Consequently, the analysis of factors affecting teicoplanin concentrations aims to provide evidence for the personalized treatment of PDAP and further improve patient outcomes, enhancing the quality of life for PD patients.

## Data Availability

The raw data supporting the conclusions of this article will be made available by the authors, without undue reservation.
