# Supplementary material for: An investigation into the correlation between intraperitoneal teicoplanin concentrations and treatment outcomes in peritoneal dialysis-associated peritonitis
Source: Front Pharmacol. 2024 Sep 9;15:1446774. doi: 10.3389/fphar.2024.1446774 (PMC11416981; doi:10.3389/fphar.2024.1446774)
Supplement: Supplementary file 1 [file DataSheet1.docx]

**Supplement data**

**Results**

**Establishment of standard curves**

Working solutions for quality control containing teicoplanin at various concentrations of 6.25, 12.5, 25, and 50 μg/mL are accurately prepared. Using multiple 1.5 mL centrifuge tubes, 360 μL of bovine serum is added to each tube. Subsequently, 40 μL of the respective standard curve series working solution, 30 μL of the internal standard solution, and 600 μL of acetonitrile are added. The contents are thoroughly mixed by vortexing, centrifuged at 14,000 rpm for 8 minutes, and 100 μL of the supernatant is transferred to sample vials for HPLC analysis.

Plot the teicoplanin standard curve sample's labeled concentration (μg/mL) on the X-axis and the ratio of the analyte's peak area to the internal standard on the Y-axis. Perform linear regression fitting using the weighted (1/X^2^) least squares method, resulting in the standard curve regression equation y = 0.0262x-0.0124 (*R^2^* = 0.9994). The linear relationship for teicoplanin concentration in PD fluid is good within the range of 6.25 to 50 μg/mL, with a lower limit of quantification at 6.25 μg/mL. The standard curve is shown in Figure S1.


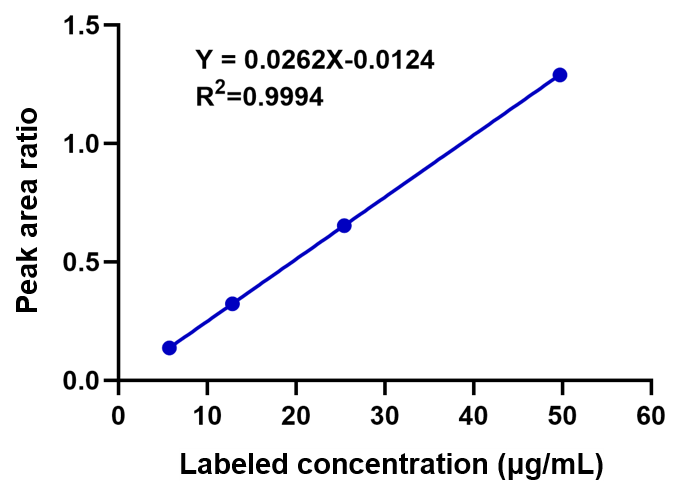


Figure S1. Standard graph of teicoplanin

**Liquid chromatography of teicoplanin**

Under the chromatographic conditions, teicoplanin and the internal standard show extended retention times, outstanding peak shapes, and no interference from sample impurities in determining methyl hydroxybenzoate in PD fluid samples, as demonstrated in Figure S2.
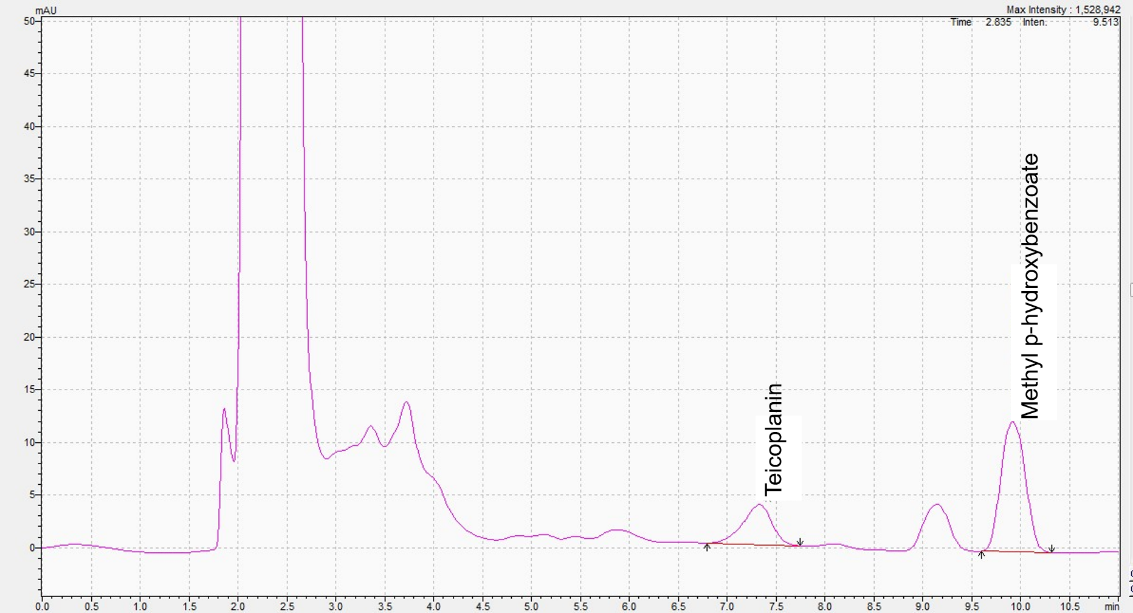


Figure S2. Liquid chromatogram of teicoplanin and methyl p-hydroxybenzoate of internal standard

**Correlation analysis between serum teicoplanin concentration and teicoplanin concentration in PD fluid**

Significant correlations were found between teicoplanin concentrations in peritoneal effluent at different time points. A significant positive correlation was observed between PDE concentrations on the 3rd day and 5th day post-medication (*r* = 0.567 > 0), between PDE concentrations on the 3rd day and 7th day post-medication (*r* = 0.626 > 0), and between PDE concentrations on the 5th day and 7th day post-medication (*r* = 0.807 > 0). In summary, there was no statistically significant correlation between serum teicoplanin concentration and PDE concentration, but a statistically significant correlation did exist among PDE concentrations (Table S1).

Table S1. Correlation analysis of serum teicoplanin concentration and PDE levels of teicoplanin

|  | Serum teicoplanin concentration | PDE-Day 1 | PDE-Day 3 | PDE-Day 5 | PDE-Day 7 |
| --- | --- | --- | --- | --- | --- |
| Serum teicoplanin concentration | 1 |  |  |  |  |
| PDE-Day 1 | -0.224 | 1 |  |  |  |
| PDE-Day 3 | 0.012 | 0.285 | 1 |  |  |
| PDE-Day 5 | - | 0.335 | 0.567** | 1 |  |
| PDE-Day 7 | - | 0.000 | 0.626** | 0.807** | 1 |

Note: *indicates statistical difference *P* < 0.05, ** indicates statistical difference *P* < 0.01.

**Comparison of PDE concentration** **compliance rates between two groups of patients**

To further assess the PDE concentration achievement rate meeting the standard between the cured and uncured groups, concentrations above 10 mg/L were considered standard. Differences in achievement rates at various time points were compared. Results indicated no significant difference in achievement rates between the groups on the first and third days post-medication. On the fifth day, 21 patients in the cured group (72.4% achievement rate) met the standard, whereas only 1 patient in the uncured group (14.3% achievement rate) did, showing a significant difference. By the seventh day, 15 patients in the cured group (51.7% achievement rate) and none in the uncured group met the standard, demonstrating a statistically significant difference (Table S2).

Table S2. Comparison of the compliance rate of PDE teicoplanin concentration at different times after intraperitoneal administration

| Time | Groups | PDE concentration compliance rate (effective/total cases) | *P* value |
| --- | --- | --- | --- |
| Day 1 | Cured Group | 19/29 (65.5%) | 0.679 |
|  | Uncured Group | 4/7 (57.1%) |  |
| Day 3 | Cured Group | 26/29 (89.7%) | 0.211 |
|  | Uncured Group | 5/7 (71.4%) |  |
| Day 5 | Cured Group | 21/29 (72.4%) | 0.030* |
|  | Uncured Group | 1/7 (14.3%) |  |
| Day 7 | Cured Group | 15/29 (51.7%) | 0.013* |
|  | Uncured Group | 0/7 (0%) |  |

Note: * indicates statistical difference *P* < 0.05.

**Comparison of Kidney Function in Patients Before and After Treatment**

When comparing renal function indicators like Scr, eGFR, Urea, UA and ALB before and after treatment in patients, it was noted that the patients' renal function remained relatively stable during the teicoplanin treatment. There were no significant changes in indicators such as Scr after medication compared to before treatment, and there was no significant difference in the patients' renal function (Table S3).

Table S3. Comparison of renal function before and after the patient's treatment

| Indicators | Pre-treatment group  (n = 36) | Post-treatment group (n = 36) | t/Z | *P* value |
| --- | --- | --- | --- | --- |
| Scr (μmol/L) | 772.45 ± 320.15 | 739.46 ± 312.14 | 0.418 | 0.678 |
| eGFR (mL/min/1.73m^2^) | 6.35 (4.72, 8.52) | 7.30 (4.90, 8.50) | -0.434 | 0.664 |
| Urea (mmol/L) | 16.20（12.55, 21.46） | 16.00 (10.20, 21.30) | -0.612 | 0.541 |
| UA (μmol/L) | 340.9 ± 114.40 | 333.82 ± 101.23 | 0.261 | 0.795 |
| ALB (g/L) | 28.66 ± 4.99 | 28.55 ± 5.97 | 0.062 | 0.951 |

Note: * indicates statistical difference *P* < 0.05.

**Comparison of other important indicators before and after treatment**

During the treatment, the patient's blood lipid levels did not fluctuate significantly. TC, TG, LDL, and HDL levels showed no notable differences pre- and post-treatment. Ca, P, and Na electrolyte levels also exhibited no significant changes. However, the potassium (K) level increased from 3.38 ± 0.66 mmol/L before treatment to 3.84 ± 0.84 mmol/L after teicoplanin anti-infection treatment, with a statistically significant difference (Table S4).

Table S4. Comparison of other important indicators before and after the patient's treatment

| Indicators | Pre-treatment group  (n = 36) | Post-treatment group (n = 36) | t/Z | *P* value |
| --- | --- | --- | --- | --- |
| TC (mmol/L) | 3.49 ± 1.02 | 2.97 ± 0.85 | 1.330 | 0.191 |
| TG (mmol/L) | 1.25 (1.02, 1.74) | 1.21 (1.01, 2.27) | -0.228 | 0.819 |
| LDL (mmol/L) | 1.91 ± 0.81 | 1.49 ± 0.69 | 1.334 | 0.189 |
| HDL (mmol/L) | 0.88 ± 0.38 | 0.76 ± 0.55 | 0.598 | 0.563 |
| Ca (mmol/L) | 2.02 (1.86, 2.18) | 2.08 (1.81, 2.26) | -0.436 | 0.663 |
| K (mmol/L) | 3.38 ± 0.66 | 3.84 ± 0.84 | -2.485 | 0.016* |
| P (mmol/L) | 1.42 (1.22, 1.79) | 1.33 (1.05, 1.64) | -1.432 | 0.152 |
| Na (mmol/L) | 134.75 ± 5.25 | 136.40 ± 5.49 | -1.265 | 0.210 |

Note: *indicates statistical difference *P* < 0.05.

**Relationship between Day-5 PDE teicoplanin Concentration and Age**

The 36 PDAP patients who received intraperitoneal administration of teicoplanin were divided into four groups based on age: ≤ 48 years, 49 ~ 61 years, 62 ~ 71 years, and > 71 years. Comparison of PDE teicoplanin concentrations between the groups showed no significant differences in concentration among different age groups (Table S5).

Table S5. Results of day-5 PDE teicoplanin concentration in different age groups

| Grouping by age | PDE teicoplanin concentration (mg/L) ($\bar{\chi}$ ± SD) |
| --- | --- |
| 1.0 (*n* = 9) | 19.46 ± 10.28 |
| 2.0 (*n* = 9) | 10.18 ± 1.68 |
| 3.0 (*n* = 9) | 20.19 ± 8.62 |
| 4.0 (*n* = 9) | 29.46 ± 12.29 |
| *F* | 0.914 |
| *P* value | 0.489 |

Note: **P* < 0.05, 1 represents ≤ 48 years old, 2 represents 49 ~ 61 years old, 3 represents 62 ~ 71 years old, and 4 represents > 71 years old.

**Relationship between day-5 PDE teicoplanin Concentration and Gender**

All patients were grouped by gender - male and female, and the PDE teicoplanin levels in both groups were compared. The analysis revealed no significant variance in teicoplanin concentrations between the genders (Table S6).

Table S6. Results of day-5 PDE teicoplanin concentrations in different genders

| Grouped by gender | PDE **t**eicoplanin concentration (mg/L) ($\bar{\chi}$ ± SD) |
| --- | --- |
| 1.0 (*n* = 22) | 19.25 ± 7.89 |
| 2.0 (*n* = 14) | 18.22 ± 6.29 |
| *t* | - 0.281 |
| *P* value | 0.678 |

Note: **P* < 0.05, 1 represents males, and 2 represents females.

On the 5th day of the study regarding the relationship between PDE teicoplanin concentration and body weight in PDAP patients undergoing intraperitoneal teicoplanin, the patients' weights were categorized into four groups: ≤ 53 kg, 54 ~ 76.5 kg, and ≥ 76.5 kg. The analysis showed no significant variance in PDE teicoplanin concentrations among the four weight groups (Table S7).

Table S7. Results of day-5 teicoplanin concentrations of PDE in different body weights

| grouping by weight | PDE teicoplanin concentration (mg/L) ($\bar{\chi}$ ± SD) |
| --- | --- |
| 1.0 (*n* = 10) | 24.23 ± 7.34 |
| 2.0 (*n* = 9) | 20.19 ± 6.74 |
| 3.0 (*n* = 8) | 15.37 ± 6.39 |
| 4.0 (*n* = 9) | 19.27 ± 7.38 |
| *F* | 0.378 |
| *P* value | 0.568 |

Note: **P*< 0.05, 1 is ≤ 53 kg, 2 is 54-60 kg, 3 is 61-76.5 kg, and 4 is ≥ 76.5kg.

**The relationship between the day-5 concentration of teicoplanin in PDE and the properties of dialysate**

PD fluid is categorized into G1.5% and G2.5% solutions, which differ in calcium content. Patients utilize PD solutions with varying concentrations based on their peritoneal function. Consequently, patients are divided into three groups: those exclusively using G1.5% low-calcium PD fluid, those solely utilizing G2.5% low-calcium PD fluid, and those employing a combination of both G1.5% and G2.5% low-calcium PD fluids. An analysis of PDE teicoplanin concentration levels among these groups indicated no statistically significant differences (Table S8).

Table S8. The results of day-5 teicoplanin concentration in PDE with varying peritoneal permeability properties

| Grouping by nature of PD fluid | PDE teicoplanin concentration (mg/L) ($\bar{\chi}$ ± SD) |
| --- | --- |
| 1.0 (*n* = 10) | 19.24 ± 10.23 |
| 2.0 (*n* = 18) | 24.48 ± 9.23 |
| 3.0 (*n* = 8) | 19.47 ± 9.45 |
| *F* | 0.342 |
| *P* value | 0.738 |

Note: **P* < 0.05, 1 refers to using only G1.5% low calcium peritoneal dialysate, 2 refers to using only G2.5% low calcium peritoneal dialysate, and 3 refers to using both G1.5% and G2.5% low calcium peritoneal dialysate simultaneously.

**The relationship between the concentration of day-5 teicoplanin in PDE and fibrinogen**

Using the quartile method, patients were classified into four groups according to varying levels of fibrinogen content: ≤ 4.1 g/L, 4.2-5.2 g/L, 5.3-6.54 g/L, and ≥ 6.55 g/L. When examining the concentrations of PDE drugs in patients with different fibrinogen content, no statistically significant differences were observed in PDE drug concentrations among the various groups (Table S9).

Table S9. Results of day-5 PDE-teicoplanin concentrations at various levels of fibrinogen content

| Grouping by fibrinogen | PDE teicoplanin concentration (mg/L) ($\bar{\chi}$ ± SD) |
| --- | --- |
| 1.0 (*n* = 9) | 30.56 ± 12.32 |
| 2.0 (*n* = 9) | 20.72 ± 8.16 |
| 3.0 (*n* = 7) | 17.35 ± 6.45 |
| 4.0 (*n* = 11) | 14.87 ± 7.07 |
| *F* | 1.456 |
| *P* value | 0.143 |

Note: **P* < 0.05, 1 represents ≤ 4.1 g/L, 2 represents 4.2 to 5.2 g/L, 3 represents 5.3 to 6.54 g/L, and 4 represents ≥ 6.55 g/L.

**The relationship between the day-5 concentration of teicoplanin in PDE and residual renal function**

The eGFR is an important basis for staging chronic kidney disease (CKD) and is the primary indicator for assessing a patient's remaining renal function. Patients' eGFR levels were grouped according to quartiles: Group 1 with eGFR ≤ 4.725 mL/min, Group 2 with eGFR 4.726 ~ 6.35 mL/min, Group 3 with eGFR 6.36 ~ 8.524 mL/min, and Group 4 with eGFR ≥ 8.525 mL/min. When comparing the differences in PDE drug concentration levels between the different groups, the results indicate that there is no statistically significant difference between the groups (Table S10).

Table S10. Results of day-5 PDE teicoplanin concentration at various levels of residual renal function

| Grouped by eGFR | PDE teicoplanin concentration (mg/L) ($\bar{\chi}$ ± SD) |
| --- | --- |
| 1.0 (*n* = 9) | 16.05 ± 5.28 |
| 2.0 (*n* = 9) | 28.39 ± 11.47 |
| 3.0 (*n* = 9) | 18.26 ± 7.45 |
| 4.0 (*n* = 9) | 21.21 ± 9.21 |
| *F* | 0.723 |
| *P* value | 0.484 |

Note: **P* < 0.05, 1 represents ≤ 4.725 mL/min, 2 represents 4.726 ~ 6.35 mL/min, 3 represents 6.36 ~ 8.524 mL/min, and 4 represents ≥ 8.525 mL/min.
